# No significantly increased frequency of the inversion polymorphism at the WBS-critical region 7q11.23 in German parents of patients with Williams-Beuren syndrome as compared to a population control

**DOI:** 10.1186/1755-8166-3-21

**Published:** 2010-11-05

**Authors:** Judith Frohnauer, Almuth Caliebe, Stefan Gesk, Carl-Joachim Partsch, Reiner Siebert, Rainer Pankau, Jutta Jenderny

**Affiliations:** 1 Institut für Humangenetik, Christian-Albrechts-Universität zu Kiel & Universitätsklinikum Schleswig-Holstein-Campus Kiel-, Schwanenweg 24, 24105 Kiel, Germany; 2 Endokrinologikum Labore Hamburg, Lornsenstr. 4-6, 22767 Hamburg, Germany; 3 Finkelstein-Klinik für Kinder-und Jugendmedizin, Heidekreis-Klinikum GmbH, Robert-Koch-Straße 4, 29664 Walsrode, Germany; 4 Labor Lademannbogen, Professor Rüdiger Arndt Haus, Lademannbogen 61-63, 22339 Hamburg, Germany

## Abstract

**Background:**

Typical Williams-Beuren syndrome (WBS) is commonly caused by a ~1.5 Mb - ~1.8 Mb heterozygous deletion of contiguous genes at chromosome region 7q11.23. The majority of WBS cases occurs sporadically but few familial cases of autosomal dominant inheritance have been reported. Recent data demonstrated the existence of the paracentric inversion polymorphism at the WBS critical region in 7q11.23 in some of the progenitors transmitting the chromosome which shows the deletion in the affected child. In parents having a child affected by WBS the prevalence of such a structural variant has been reported to be much higher (~25- ~30%) than in the general population (~1- ~6%). However, in these previously reported studies only a limited number of randomly selected patients and non transmitting parents of WBS patients were used as controls, but without specification of any clinical data. Therefore we have undertaken a German population-based molecular cytogenetic investigation. We evaluated the incidence of the paracentric inversion polymorphism at 7q11.23 analyzing interphase nuclei of lymphocytes using a three color fluorescence in situ hybridization (FISH) probe.

**Results:**

FISH analysis was carried out on couples with a child affected by WBS as compared to a population sample composed of different normal individuals: Control group I: couples with two healthy children, control group II: couples with fertility problems, planning ICSI and control group III: couples with two healthy children and one child with a chromosome aberration, not involving region 7q11.23. The three color FISH assay showed that the frequency of the paracentric inversion polymorphism at 7q11.23 in couples with a child affected by WBS was 20.8% (5 out of 24 pairs) as compared to 8.3% (2 out of 24 pairs, control group I), 25% (4 out of 16 pairs, control group II) and 9.1% (1 out of 11 pairs, control group III), respectively (total 7 out of 51 pairs, 13.8%). The frequencies differed between the groups, but this was statistically not significant (p > 0.05, Fisher's test).

**Conclusion:**

Our results do not support the hypothesis that the paracentric inversion polymorphism at 7q11.23 is a major predisposing factor for the WBS deletion.

## Background

Williams-Beuren syndrome (WBS) is a developmental disorder with multisystemic manifestations mainly characterized by vascular stenoses (predominantly supravalvular aortic stenosis (SVAS)), distinctive craniofacial features, mental retardation with a characteristic neurocognitive profile, short stature and some endocrine and connective tissue abnormalities. The WBS syndrome is caused by a heterozygous deletion of contiguous genes at chromosomal region 7q11.23. The common deletions in WBS patients span a genomic region of ~1.5 - ~1.8 mega base pairs (Mb), encompassing approximately 28 genes [1,2].

The majority of WBS cases occur sporadically with an estimated prevalence of the disorder as high as 1/7500 newborns [3]. Few cases of autosomal dominant inheritance have been reported. In these families the disease transmitting progenitors usually presented a relatively mild phenotype [4-7].

Previous data demonstrated the existence of the paracentric inversion polymorphism in some of the progenitors transmitting the WBS chromosome [8,9]. Thereby the breakpoints of the inversion (~1.8 - ~2.6 Mb) always lie externally to the WBS single copy region (~1.2 Mb) and do not disrupt any of the actively expressed genes commonly deleted in patients affected by WBS. That is in agreement with the observed lack of clinical symptoms in almost all WBS transmitting progenitors [9].

**Figure 1:**
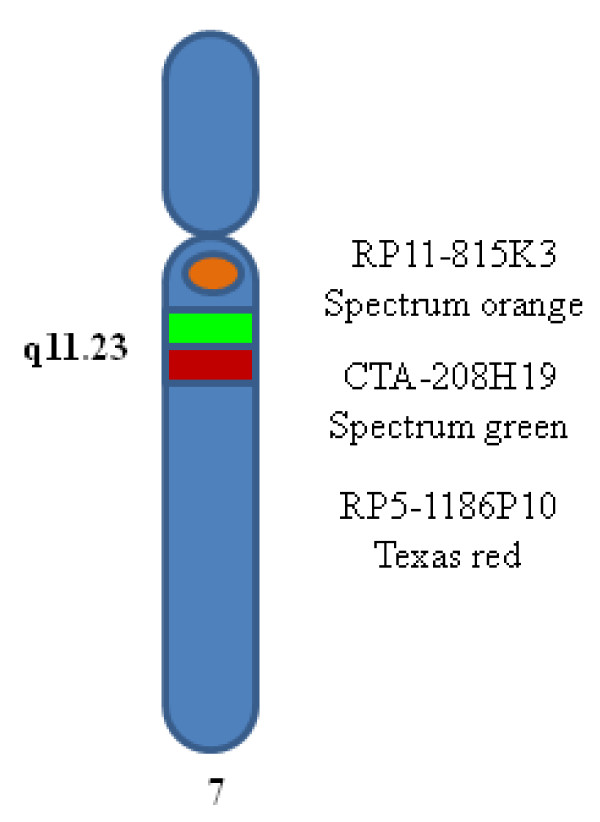
**The three color constructed FISH probe**.

Based on the results of Osborne et al. [8] and Bayés et al. [9], it is generally supposed that the prevalence of the paracentric inversion polymorphism at 7q11.23 is much higher in the population of WBS parents (~30%) than in the general population (5%) [1]. Recently, in a large-scale analysis Hobart et al. [10] studied the paracentric inversion polymorphism at 7q11.23 in 257 children with WBS and their parents by interphase FISH. After determining the parents-of-origin of the deleted chromosome by molecular genetic methods, they found that the frequency of the paracentric inversion polymorphism at 7q11.23 in the transmitting parent group was 24.9% and in the non transmitting parent group 5.8%. They concluded that the value of 5.8% represents a reasonable estimate of the rate in the normal population. However, in all previously reported studies only randomly selected patients and non transmitting parents of WBS patients were used as controls, but without specification of any clinical data. Therefore, we undertook an extensive German population-based molecular cytogenetic investigation and examined parents of patients with Williams-Beuren syndrome and well-defined control groups. We determined the frequency of the paracentric inversion polymorphism at 7q11.23 in different German cohorts analyzing interphase nuclei of lymphocytes using a three color fluorescence in situ hybridization (FISH) probe (Figure 1). We wanted to answer three questions: First, what is the frequency of the paracentric inversion polymorphism at 7q11.23 in couples with a child affected by WBS? Second, what is the frequency of the paracentric inversion polymorphism at 7q11.23 in the German population analyzing individuals from different groups? Third, vary the frequencies between selected groups for a paracentric inversion polymorphism at 7q11.23?

## Results

### Cut-off level for the frequency of the paracentric inversion polymorphism at 7q11.23

**Figure 2:**
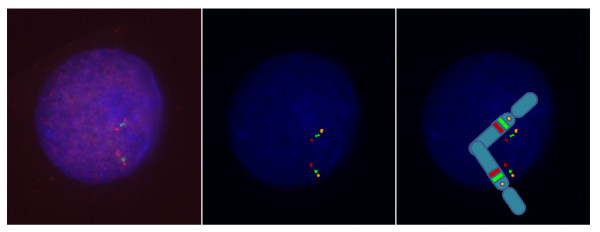
**Normal FISH signals at chromosome 7q11.23.** (left: microscope FISH image, middle: enhanced FISH image, right: drawing of the chromosome image with FISH signals).

**Figure 3:**
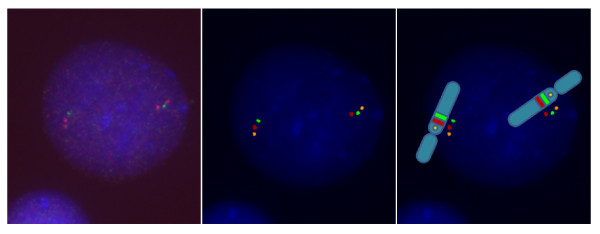
**A normal chromosome 7 and a paracentric inversion at chromosome 7q11.23.** (left: microscope FISH image, middle: enhanced FISH image, right: drawing of the chromosome image with FISH signals).

**Figure 4:**
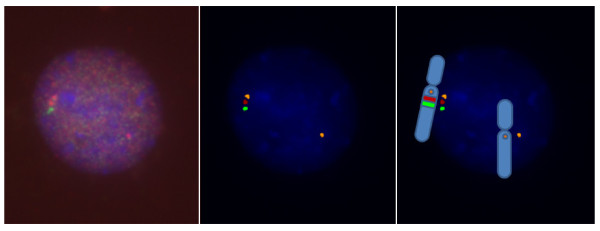
**A paracentric inversion and a deletion at chromosome 7q11.23.** (left: microscope FISH image, middle: enhanced FISH image, right: drawing of the chromosome image with FISH signals).

Based on the results from 5 controls, a test result was defined negative (no inversion carrier) when at least 81% of interphase nuclei showed the normal signal configuration. A test result was defined positive (inversion carrier) when more than 15% of interphase nuclei showed the paracentric inversion configuration "spectrum orange - Texas red - spectrum green". An example of a normal- and inverted-orientated interval segment as well as a 7q11.23 microdeletion, as detected by three color FISH, is shown in Figure 2, 3 and 4.

### Parents of patients with a Williams-Beuren syndrome

In families with a child affected by WBS the paracentric inversion polymorphism at 7q11.23 was documented by three color FISH in 5 out of 24 couples (20.8%). Five males and one female were found to be carrier of the paracentric inversion polymorphism at 7q11.23. In one couple both the father and the mother carried the polymorphic variant (Table 1).

**Table 1:** Clinical data of patients and FISH results

Samples	Parameters for FISH analyses	n	With inversion	With inversion %	P-value	Female carrier	Male carriers
WBS parents II	Couples with a WBS child	24	5	20.8		1*	5*

Control group I	Couples with two healthy children	24	2	8.3	0.42	0	2

Control group II	Couples with fertility problems, planning ICSI	16	4	25.0	1.00	1	3

Control group III	Couples with two healthy children and one child with a chromosome aberration (region 7q11.23 not involved)	11	1	9.1	0.64	1	0

Control groups (I-III)		51	7	13.7		2	5

						3	10

*In one couple both of the father and the mother carried a paracentric inversion polymorphism at 7q11.23

### WBS patients of families having the paracentric inversion polymorphism at 7q11.23

Using the three color FISH probe we consistently detected the deletion at 7q11.23 in all affected WBS patients who were studied. Analyzing the other chromosome 7, they showed a normal signal configuration, indicating a normal homologous chromosome 7. The only exception was one WBS patient in whom the paracentric inversion polymorphism at 7q11.23 was detected on the non-deleted chromosome 7 in 60% nuclei analyzed. The inversion was of paternal origin. The mother had had a normal signal configuration in 90% of the nuclei analyzed.

### Control groups

In the control sample, 7 out of 51 couples (13.7%) were carriers of the paracentric inversion polymorphism at 7q11.23. In one further proband we detected 24 interphase nuclei with normal signals and 23 interphase nuclei demonstrating the inversion pattern. This result could possibly be consistent with mosaicism for the polymorphic inversion. However, as a precaution this case was formally classified as normal.

The paracentric inversion polymorphism at 7q11.23 was found in 8.3% of couples with two healthy children (control group I, 2 out of 24), in 25% of couples with fertility problems, planning ICSI (control group II, 4 out of 16) and in 9.1% of couples with two healthy children and one child with a chromosome aberration (not involving region 7q11.23) (control group III, 1 out of 11). In control group II, 3 out of 4 carriers were infertile male patients with azoospermia/oligoasthenozoospermia. The female carrier was suspected to suffer from blocked fallopian tubes. The frequencies differed between the three subgroups, but this was statistically not significant (p > 0.05, Fisher's test)(Table 1).

## Discussion

For diagnostic purpose, FISH analysis with commercial single FISH probes is widely used to detect WBS deletions at 7q11.23. Our experimental three color FISH probe that was used consists of two DNA probes covering the WBS critical region and accurately detects all previously diagnosed deletions in WBS patients of families who have one parent carrying the paracentric inversion polymorphism.

The three color FISH assay was a powerful tool for the detection of the paracentric inversion polymorphism at 7q11.23 in interphase nuclei. Taking together all FISH data, the frequency of the polymorphic variant in couples with a child affected by WBS was 20.8% (5 out of 24) versus 8.3% (2 out of 24, control group I), 25% (4 out of 16, control group II) and 9.1% (1 out of 11, control group III), respectively.

In contrast to previous studies we have been unable to find evidence of a statistically increased frequency of the paracentric inversion polymorphism at 7q11.23 exclusively within the WBS population. Moreover, we found a high frequency of this structural variant in the German population sample composed of different groups of normal individuals. Therefore the previously reported results need to be interpreted with caution: Using interphase FISH and polymorphic DNA marker analysis, Osborne et al. [8] observed the paracentric inversion polymorphism at 7q11.23 solely in the progenitors transmitting the WBS chromosome. Almost one third (33%, 4 out of 12) of WBS parents were found to be heterozygous for this polymorphic variant. As controls Osborne et al. [8] used randomly selected blood samples (n = 26) from ethnically diverse sources and 16 non transmitting parents of WBS patients, but without specification of any clinical data. By site-specific nucleotide (SSN) assays Bayés et al. [9] predicted that 28% (21 out of 72) of the WBS transmitting progenitors were heterozygous for the paracentric inversion polymorphism at 7q11.23, but using three color interphase FISH confirmation was only obtained in four parental genomes (5.5%). This rate was similar to the degree of polymorphic variations at 7q11.23 in the control group (5.7%). Hobart et al. [10] investigated the inversion status in 257 children with WBS and their parents. They evaluated that the paracentric inversion at 7q11.23 might be present in about 5.8% in the general population. However, similar to Osborne et al. [8], they calculated their risk estimation on data obtained from the non transmitting parent group. Our three color FISH results indicate that the paracentric inversion polymorphism at 7q11.23 is not a necessary factor for the WBS deletion to occur. Furthermore, the presence of the paracentric inversion polymorphism on the non-deleted chromosome 7 in one WBS patient indicates that the parental genome probably did not contribute to the disease-related chromosome.

In couples with fertility problems, planning ICSI, 4 probands showed the paracentric inversion polymorphism at 7q11.23. Three were male carrier, suffering from azoospermia/oligoastenozoospermia. The WBS deletion includes the gene *FKBP6 *which has recently been shown to play a role in male reproduction, showing azoospermia in knockout mice [11]. In humans mutations in the *FKBP6 *gene does not appear to cause azoospermia [7,12]. In a larger sample based on 323 infertile patients, Zhang et al. [13] observed a significantly decrease of individual haplotypes for the *FKBP6 *gene in the disease related group compared with controls. Therefore, an association of the *FKBP6 *gene with human male infertility cannot be excluded.

Presence of the paracentric inversion polymorphism predisposes to chromosomal mispairing. The classical theory is that a carrier with such a structural variant cannot produce viable progeny [14]. If a recombinant event is formed during meiosis following a crossover within the inverted segment, this would result in either an acentric or a dicentric chromosome with a high rate for fetal loss. However, in the study of Hobart et al. [10], the incidence of early abortions among the WBS and non WBS population was not increased, indicating that the paracentric inversion polymorphism at 7q11.23 is not associated with a particular risk of miscarriage.

We found an higher frequency of male inversion carriers (10 out of 75 males versus 3 out of 75 females), but this was statistically not significant (p-value 0,07). Osborne et al. [8] found a higher rate of female inversion carriers in 12 families with a child affected by WBS (one male versus three females). In the large-scale analysis of Hobart et al. [10] they did not find any differences in the rate of maternal versus paternal inversion status with the mother in 14.4% (37 out of 257) and the father in 16.3% (42 out of 257) of couples.

The mechanism involved in formation of WBS deletions is not yet clear. The vast majority of WBS deletions is of similar size and has tightly clustered breakpoints. Therefore, it seems likely that it is the unique genetic architecture in the WBS region, that might be responsible for the occurrence of genomic rearrangements: The WBS region consists of a single copy gene region (~1.2 Mb) flanked by three large blocks of DNA that have a very high degree of similarity to one another (called low copy repeats (LCRs) or duplicons). Regions enriched in LCRs are known to show higher genomic instability mediated by unequal crossing over (non allelic homologous recombination (NAHR)) during meiosis or mitosis. The architecture of LCRs and underlying mechanisms have been investigated in different genomic disorders. Directly oriented LCRs are likely to result either in a deletion or a reciprocal duplication, whereas inverted repeats lead to an inversion of a DNA segment between the LCRs [8,15]. Indeed, in addition to the WBS deletion, patients with a duplication of the WBS region have been observed especially since the introduction of molecular karyotyping/array-CGH. These patients do not physically or cognitively resemble patients with a WBS deletion. They display facial dysmorphism with straight eyebrows, a high broad nose, and a thin vermilion of the upper lip. The most striking feature is a severe delay in expressive speech [16-18]. Large copy number variants (CNVs) are significantly enriched in regions with LCRs. The rate of CNVs is up to 4-fold higher in WBS transmitting progenitors as compared to controls, that additionally may act as a susceptibility factor for the WBS deletion to occur [19].

## Conclusions

The incidence of the paracentric inversion polymorphism at 7q11.23 in the general population varies between different studies. Further experiments are required to determine why the discrepancies exist. Therefore, a larger German study should be conducted to support our results. However, it now seems likely that WBS is caused by genomic rearrangement mostly mediated by NAHR, but not by the paracentric inversion polymorphism at 7q11.23.

## Methods

### Parents of patients with a Williams-Beuren syndrome and control groups

To detect the frequency of the paracentric inversion polymorphism at 7q11.23 in WBS families, a total of 24 couples having a child with WBS were analyzed in the framework of the German Bundesverband Williams-Beuren-Syndrom e.V.. We analyzed individuals affected by WBS and their parents as well as control groups from the Department of Pediatrics and Institute of Human Genetics of the University of Kiel and families counseled by R.P.. In the individuals affected by WBS the clinical diagnosis had been confirmed by FISH using commercial DNA probes.

To detect the incidence of the paracentric inversion polymorphism at 7q11.23 in the German population, a cohort composed of different normal individuals were used as control: Control group I consisted of couples with two healthy children, control group II of couples with fertility problems, planning ICSI, and control group III of couples with two healthy children and one child with a chromosome aberration, not involving region 7q11.23. The parents with a child affected by WBS and parents with two healthy children provided informed consent to R.P. Controls included left-over materials from diagnostic chromosome analyses studied in an anonymised fashion in compliance with the rules of the local IRB. The detailed description and the number of individuals in the different groups are given in Table 1.

### FISH analysis

Cytogenetic preparations were made by standard method from peripheral blood lymphocytes. A three color FISH probe was constructed: FISH was performed with one BAC located proximal to the critical WBS region (AC005074.1, 71641293 basepairs (bp)) from pter, region 7q11.22, Invitrogen, Karlsruhe, Germany), and two BACs within the critical WBS region 7q11.23 (AC005074.1, 72850847 bp from pter, Invitrogen, Karlsruhe, Germany, and AC0052313, 73992744 bp from pter, Welcome Trust Sanger Institute, Cambridge, England) (http://genome.ucsc.edu. Febr. 2009 (GRCh/hg19, viewed in July 2010), labeled with spectrum orange, spectrum green and Texas red, respectively (Figure 1)). Purified BAC and PAC DNAs were labeled with fluorescent dyes by random priming using BioPrime^® ^DNA Labeling System (Invitrogen, Karlsruhe, Germany). Unincorporated nucleotides were removed by Microcon^® ^Ultracel YM-30 Centrifugal Filter Units (Millipore GmbH, Schwalbach, Germany). Hybridization was performed by use of standard protocols. Nuclei were counterstained with DAPI/antifade (Serva, Heidelberg, Germany). When using two DNA probes from the WBS commonly deleted region and one DNA probe centromeric to the deletion, it is possible to decide whether the order of the signals in one chromosome 7 is normal (Figure 2). A different order (spectrum orange - Texas red - spectrum green) in the other chromosome 7 indicates a change (paracentric inversion) in the orientation of the WBS region (Figure 3). Loss of signals (spectrum green - Texas red) is seen in a deletion (Figure 4). Slides were visualized under a fluorescent microscope (Axioskop, Carl Zeiss Microimaging GmbH, Oberkochen, Germany) and images were analyzed with the ISIS V 5.2.11 software (MetaSystems GmbH, Altlussheim, Germany). The three color FISH probe was tested on lymphocytes of 5 controls with a normal karyotype. 100 interphase nuclei per case in which all three DNA probes could be identified on both chromosomes 7 in close alignment with each other were scored. Cut-off levels for the definition of the paracentric inversion at 7q11.23 were derived from this analysis. The mean value of 89.6% with three standard deviations (2.88%) was used as cut-off. Using this formula the cut-off level was 81%. To determine the incidence of the paracentric inversion polymorphism at 7q11.23 in parents of patients affected by WBS and control groups 50 interphase nuclei per individual were examined with the three color FISH probe. For validation, 5 individuals affected by WBS with a proven deletion in 7q11.23 were additionally examined with the three color FISH probe. Fisher's exact test was applied to evaluate the differences between data of different groups. A p value < 0.05 was considered to be statistically significant. All performed tests were two-sided.

## Competing interests

The authors declare that they have no competing interests.

## Authors' contributions

JF carried out and evaluated the FISH studies, AC supervised cytogenetic studies in patients and controls, counseled patients and controls and supervised FISH experiments, SG assisted in the FISH evaluation, RS was involved in study design and FISH probe design, CJP and RP characterized the patients and provided blood samples of patients and their parents, RP counseled the parents of patients with WBS and controls, JJ performed data evaluation and drafted the paper, and all authors contributed to the finalizing, read and approved the final manuscript.
